# CD4^+^ Th1 and Th17 responses and multifunctional CD8 T lymphocytes associated with cure or disease worsening in human visceral leishmaniasis

**DOI:** 10.3389/fimmu.2024.1277557

**Published:** 2024-02-12

**Authors:** Mariana Nobre Farias de Franca, Lorranny Santana Rodrigues, Aline Silva Barreto, Geydson Silveira da Cruz, José Carlos Aragão-Santos, Angela Maria da Silva, Amélia Ribeiro de Jesus, Clarisa B. Palatnik-de-Sousa, Roque Pacheco de Almeida, Cristiane Bani Corrêa

**Affiliations:** ^1^Laboratory of Biology and Immunology of Cancer and Leishmania, Department of Morphology, Federal University of Sergipe, Aracaju, Sergipe, Brazil; ^2^Graduate Program in Health Sciences, Federal University of Sergipe, Aracaju, Sergipe, Brazil; ^3^Division of Immunology and Molecular Biology Laboratory, University Hospital/Empresa Brasileira de Serviços Hospitalares (EBSERBH), Federal University of Sergipe, Aracaju, Sergipe, Brazil; ^4^Department of Physical Education, Federal University of Sergipe, Aracaju, Sergipe, Brazil; ^5^Department of Medicine, Federal University of Sergipe, Aracaju, Sergipe, Brazil; ^6^Department of Medicine, Federal University of Sergipe, Immunology Investigative Institute (III), INCT, CNPq, Aracaju, Sergipe, Brazil; ^7^Institute of Microbiology Paulo de Góes, Federal University of Rio de Janeiro, Immunology Investigative Institute (III), Insititutos nacionais de Ciência e Tecnologia (INCT), Conselho Nacional de Desenvolvimento Científico e Tecnológico (CNPq), Rio de Janeiro, Brazil; ^8^Graduate Program in Vegetal Biotechnology and Bioprocesses, Federal University of Rio de Janeiro, Rio de Janeiro, Brazil

**Keywords:** cellular immunity, immunophenotyping, T-cell subsets, multifunctional, human visceral leishmaniasis

## Abstract

**Introduction:**

In VL, a proinflammatory phenotype is typically associated with enhanced phagocytosis and a Th1 mediated immune response resulting in infection control. In contrast, an anti-inflammatory phenotype, associated with a predominant regulatory response, typically enables intracellular multiplication of *Leishmania* parasites and disease progression.

**Methods:**

To investigate the impact of chemotherapy on Th2 and Th17 immune responses in patients with visceral leishmaniasis (VL), we assessed all combinations of intracellular expression of IFN-γ, IL-10, IL-4 and IL-17 in the CD4^+^ and CD8^+^ T cell populations of peripheral blood mononuclear cell (PBMC) samples from patients, after antigenic stimulation with *Leishmania* lysate, throughout treatment and follow-up. As increases in spleen and liver sizes and decreases in hematocrit, hemogloblin, erythrocytes, monocytes, leukocytes and platelets levels are strongly related to the disease, we studied the correlations between the frequencies of T cells producing the afore mentioned cytokines, individually and in combination, and these variables, as markers of disease or cure.

**Results:**

We found that the frequency of IFN-γ-producingCD4^+^ T cells increased until the end of chemotherapy with Glucantime® or AmBisome ®, while IL-10, IL-4 and IL-17-producing CD4^+^ T cells peaked on day 7 following the start of treatment. Although the frequency of CD4^+^IL-17^+^ cells decreased during treatment an increase was observed after clinical cure. The frequency of CD4^+^ T cells producing only IFN-γ or IL-17 correlated with blood monocytes levels. Frequencies of double-producers of IFN-γ and IL-10 or IL-4 correlated positively with eosinophils and platelets levels. Together, this suggest that IFN-γ drives the immune response towards Th1 at cure. In contrast, and associated with disease or Th2 response, the frequency of CD4^+^ IL-10^+^ cells correlated positively with spleen sizes and negatively with circulating monocyte levels, while the frequency of CD4^+^ producing both IL-4 and IL-10 correlated negatively with platelets levels. The frequency of CD8^+^ single-producers of IFN-γ increased from day 21 to 90 while that of single-producers of IL-10 peaked on day 7, of IL-4 on day 30 and of IL-17, on day 180. IFN-γ expression in CD8^+^ single- and double-producers of cytokines was indicative of an immune response associated with cure. In contrast, frequencies of CD8^+^ double-producers of IL-4 and IL-10, IL-4 and IL-17 and IL-10 and IL-17 and producers of three and four cytokines, were associated with disease and were low after the cure. Frequencies of CD8^+^ T cells producing IFN-γ alone or with IL-17 were positively correlated with platelets levels. In contrast, as markers of disease: 1) frequencies of single producers of IL-10 correlated negatively with leukocytes levels, 2) frequencies of double producers of IL-4 and IL-10 correlated negatively with platelet, leukocyte, lymphocyte and circulating monocyte levels, 3) frequencies of triple-producers of IFN-γ, IL-4 and IL-10 correlated negatively with platelet, leukocyte and neutrophil levels and 4) frequencies of producers of IFN-γ, IL-4, IL-10 and IL-17 simultaneously correlated positively with spleen size, and negatively with leukocyte and neutrophil levels.

**Discussion:**

Our results confirmed that the clinical improvement of VL patients correlates with the decrease of an IL-4 and IL-10 CD4^+^Th2 response, the recovery of CD4+ Th1 and Th17 responses and the frequency of CD8^+^ single-producers of IFN-γ and double producers of IFN-γ and IL-17.

## Introduction

Visceral leishmaniasis (VL) is a severe protozoan infection caused by species from the *Leishmania* genus (1). The global estimate of new VL cases is 50,000 to 90,000 annually, with Brazil, India, and East Africa reporting the highest number of cases (2). VL is also endemic in Iran and Iraq. Also known as kala-azar, this disease is the most lethal form of the three clinical forms of leishmaniasis (cutaneous, mucocutaneous, and visceral), leading to death in 95% of cases that are left untreated (3, 4). The spectrum of clinical manifestation in leishmaniasis depends on the characteristics of the parasite and the host, such as its immune status. In VL, the main symptoms are high fever, splenomegaly, hepatomegaly, anemia, weight loss, hypergammaglobulinemia, and progressive suppression of the cellular immune response (5, 6).

An effective immune response against leishmaniasis depends on cell-mediated immunity. CD4^+^ and CD8^+^ T cells, as well as cells of the innate immune response, such as macrophages, are deeply involved in disease outcome, as in the pattern of cytokines released during infection. Different subsets of CD4^+^ T cells exist, such as T helper (Th) 1 type, Th2, Th17, and regulatory T cells (T reg), which produce cytokines and chemokines that recruit and activate cells (7). In fact, the T-cell response and cytokine profile change throughout the course of infection, and as such, the correlation between the Th1/Th2 response in leishmaniasis has been extensively studied (8).

In VL, proinflammatory cytokines, such as TNF-α and IL-12, are important to trigger a Th1 response, inducing IFN-γ production and leading to parasitic clearance as a consequence of the generation of reactive oxygen species (ROS) (8). Other cell-mediated Th1 response cytokines, IL-2, IL-1β, IL-22, IL-23, and IL-18, have frequently been associated to the host’s protective response (9–11). Seder and colleagues (2008) proposed a model for the development of the CD4^+^ Th1 response in leishmaniasis, which involved the expression of different combinations of three proinflammatory cytokines: IL-2, TNF-α, and IFN-γ (10). In this model, after contact with an antigen, a CD4 naïve cell can become single-producers of IL-2 or TNF-α, followed by double-producers of IL-2 and TNF-α and finally, triple-producers of IL-2, TNF-α, and IFN-γ, which are then the central memory cells (T_CM_) that are long-lasting, able to proliferate, and show effector capabilities in a new meeting with the parasite. These T_CM_ cells can further differentiate into CD4 T cells secreting TNF-α and IFN-γ, then IL-2 and IFN-γ, and finally, single-producers of IFN-γ, which are effector cells that undergo apoptosis (10). This model for the development of the CD4 T-cell response was intensively studied in leishmaniasis vaccine (10–16) and infection cure models (9) as well as in other infectious diseases (17–19).

Regarding the cure of VL, we recently described a longitudinal investigation in a cohort of patients with VL (9). Multifunctional CD4^+^ T cells that express IL-2, TNF-α, and IFN-γ were associated with cure, and CD4^+^ T cells secreting IFN-γ and TNF-α increased progressively throughout the treatment course. With regard to CD8^+^ T cells, triple-producers of IL-2, TNF-α, and IFN-γ, double-producers of TNF-α and IFN-γ, and single-producers of TNF-α were also associated with the cure in this cohort. Furthermore, the increase in CD8^High^ and the decrease in CD8^Low^ T-cell frequencies were correlated with the cure of VL (9).

Th17 cells express IL-17 and have an important modulating effect in leishmaniasis and other diseases (20, 21). They represent another subset of CD4 T cells different from Th1 and Th2, which contributes to the inflammatory response and to the resistance to infection through diverse effector functions, depending on the pathogen (20). In VL, IL-17 has been associated to asymptomatic patients (22). In filarial co-infected patients with malaria, the reduction in the Th17 response leads to ineffective T cell activity (23). Furthermore, Th17 cells are also more likely to be infected with HIV, and their suppression is present in the acute phase of the disease (24, 25). In contrast, IL-17 has also been involved with susceptibility to VL (26, 27). For example, in a mouse model of VL, IL-17 suppression contributed to an effective response against *L. donovani* infection (26, 28). This harmful role of IL-17 produced by Th17 cells was also observed in tuberculosis (21).

While the role of the multifunctional Th1 response in vaccination and treatment of leishmaniasis has been intensively investigated (9–16), the development of the multifunctional Th2 response during VL has been less studied. In the present study, we investigated the combined production of IFN-γ and the Th2 and Th17 cytokines—IL-10, IL-4, and IL-17 in CD4^+^ and CD8^+^ T cells of patients with VL throughout treatment—to evaluate the impact of the chemotherapy on this arm of the intracellular immune response toward the cure of human VL.

## Methods

### Ethical statement

This study was performed according to the standards established by the Declaration of Helsinki and followed the guidelines and regulations of the Brazilian National Council of Health resolution 196/96. The study was also approved by the Research and Ethics Committee of the Federal University of Sergipe (UFS)-University Hospital, Aracaju, Sergipe State (SE), Brazil (CAAE 0162.0.107.000-09). A written informed consent was obtained from all patients before the study.

### Patients and clinical evaluation

A total of 13 symptomatic patients with VL (five women and eight men) were included in this study. The diagnostic criteria were established according to the guidelines of the Brazilian Ministry of Health (29): clinical signs of VL (fever, hepatosplenomegaly, anemia, weight loss, spleen and liver enlargement, and pancytopenia) and positivity in the rK39 rapid test, confirmed by the presence of parasites in myelogram or in *in vitro* culture of bone marrow aspirate obtained through puncture (**Supplementary Figure S1**). Patients who tested positive for HIV infection, who showed symptoms associated to other acute or chronically infectious disease, or who used immunosuppressant drugs were excluded from the study (**Supplementary Figure S1**).

The age of the patients ranged between 20 and 59 years (mean = 39.8 years). Regarding chemotherapy, five patients received 20 mg/kg/day of meglumine antimoniate (MA) (Glucantime ® Sanofi) for an average of 21 days, while eight patients received 3 mg/kg/day of liposomal Amphotericin B (LAMB) (AmBisome®, Gilead) for 7 days (9) (**Supplementary Figure S1**). As a healthy control group, 12 individuals from the endemic area (eight women and four men), aged between 20 and 60 years, who had no history of infectious diseases and had not received immunosuppressive therapy at the time of collection were included.

Peripheral blood samples of patients and healthy controls were collected for the isolation of peripheral blood mononuclear cells (PBMC) and clinical and laboratory assessments (hemoglobin gr/dL, hematocrit %, platelets × 10^-3^, leukocytes/mm (3), neutrophils/mm (3), lymphocytes/mm (3), eosinophils/mm (3), and monocytes/mm (3)) before, during (**Supplementary Figure S1**), and after treatment (days 0, 7, 14, 21, 30, 60, 90, and 180 after the start of treatment; **Supplementary Figure S2**). Patients were considered cured after 180 days of follow-up with no relapse (29).

The hematological analysis was performed using a Cell-Dyn Ruby™ flow cytometer (Abbot, IL, USA) based on the multiangle polarized scatter separation technology (https://www.corelaboratory.abbott/int/pt/offerings/brands/cell-dyn/cell-dyn-ruby.html). Increases in the spleen and liver sizes were measured in centimeters, below the lower edge of the rib (29). The same group of patients included in this investigation was studied before for their multifunctional and CD8 High Low T-cell response. As we have described (9), all patients achieved an effective clinical cure with no relapse for at least 2 years (**Supplementary Figure S2**) (29).

### Flow cytometry analysis

PBMCs were isolated from peripheral blood samples from healthy controls (*n* = 12) and symptomatic patients (*n* = 13) by gradient centrifugation on Ficol®-Paque (Sigma-Aldrich, MO, USA). PBMCs were suspended in RPMI 1640 supplemented with 10% fetal calf serum and 1% streptomycin, then plated in 48-well plates (10 (6)/well), and centrifuged. The pellets were suspended with supplemented RPMI 1640 medium and further incubated with 10 µg/mL of soluble *L.* (*L.*) *infantum chagasi* (SLA) antigen, or not, for 18 h at 37°C and 5% CO_2_ using the GolgiPlug™ Cytokine Permeation kit (BD, Pharmingen, NJ, USA) as described previously (9). Following this, the cells were incubated with 100 µL of Fc receptor blocking solution for 20 min at 4°C. After centrifuging at 405 × *g* for 5 min, the cells were incubated with anti-human CD3 APCCy7 (Clone HIT3a, BioLegend, CA, USA), anti-human CD4 FITC (Clone RPA-T4, BD Pharmingen™, NJ, USA), and anti-human CD8 PECY 5 (Clone HIT8a, BD Pharmingen™, NJ, USA) antibodies for 30 min for surface labeling, then washed, centrifuged, permeabilized, and fixed using the Citofix/Citoperm® Fixation/Permeabilization kit (BD Biosciences, NJ, USA); fixed and further incubated with the rat anti-human IL-4 BV421 (Clone MP4-25D2, BD Horizon™, NJ, USA), anti-human IL-10 PE (Clone JES3-9D7, eBioscience™, USA), anti-human IL-17 Alexa 647 (Clone N49-653, BD Pharmingen™, NJ, USA), and anti-human IFN-γ PECy-7 (Clone B27, BD Pharmingen™, NJ, USA) antibodies, for 30 min, for the detection of intracellular cytokine expression. After this period, the cells were washed and centrifuged, and 30,000 events were acquired in the lymphocyte gate using a BD FACSCanto II® flow cytometer using the FACSDiva® software.

For the functional analysis of T-cell populations obtained by multiparametric flow cytometry, FSC-A *versus* SSC-A parameters were used to determine the lymphocyte population (**Supplementary Figure S3**). After that, CD3^+^/CD4^+^ and CD3^+^/CD8^+^ populations were selected. Within each population, cells positive for IFN-γ, IL-4, IL-10, and IL-17 were analyzed. The Boolean gate strategy was used for the combinatorial analysis of single-, double-, triple-, and quadruple-cytokine production. These data were analyzed using FlowJo^®^ v10.9 software (BD Biosciences, NJ, USA).

For this study, the population frequency (%), integrated mean fluorescence intensity (iMFI), and MFI of the cells producing each cytokine were recorded. iMFI is a widely used parameter that reflects cell frequency multiplied by MFI. Furthermore, the frequencies of lymphocytes producing each combination of two, three, and four cytokines were also recorded. For population frequency and iMFI analysis, samples of the same patient that were not treated with antigen were considered as baseline controls and these values were subtracted from the values obtained for cells treated with SLA.

### Statistical analysis

The GraphPad Prism 9 software was used to generate the graphs and for statistical analyses. Confidence interval 95% (CI 95%) was used for comparisons of total frequencies and iMFI of CD4^+^ and CD8^+^ T cells and Pearson bivariate two-tailed test for correlation analysis.

## Results

### Total frequencies and iMFIs of cytokine-producing CD4^+^ T cells

Patients with VL were considered symptomatic from day 0 (before the start of the treatment) until day 90 (D0–D90) and cured from day 180 (D180). Raw clinical data for each patient including age, period of symptoms, type and duration of treatment, time to reach clinical improvement and cure, and other clinical data used for analysis are summarized in **Supplementary Table S1**. To determine the time to clinical improvement (days), we considered the spleen size reduction to 0 cm or normalization of leukocyte counts. However, clinical cure as defined by the guidelines of the Brazilian Ministry of Health is the absence of relapse after 6 months of follow-up (180 days). The recurrence of the disease up to 12 months after treatment would be considered recidivate (29) (**Supplementary Figure S2**).

**Figure 1:**
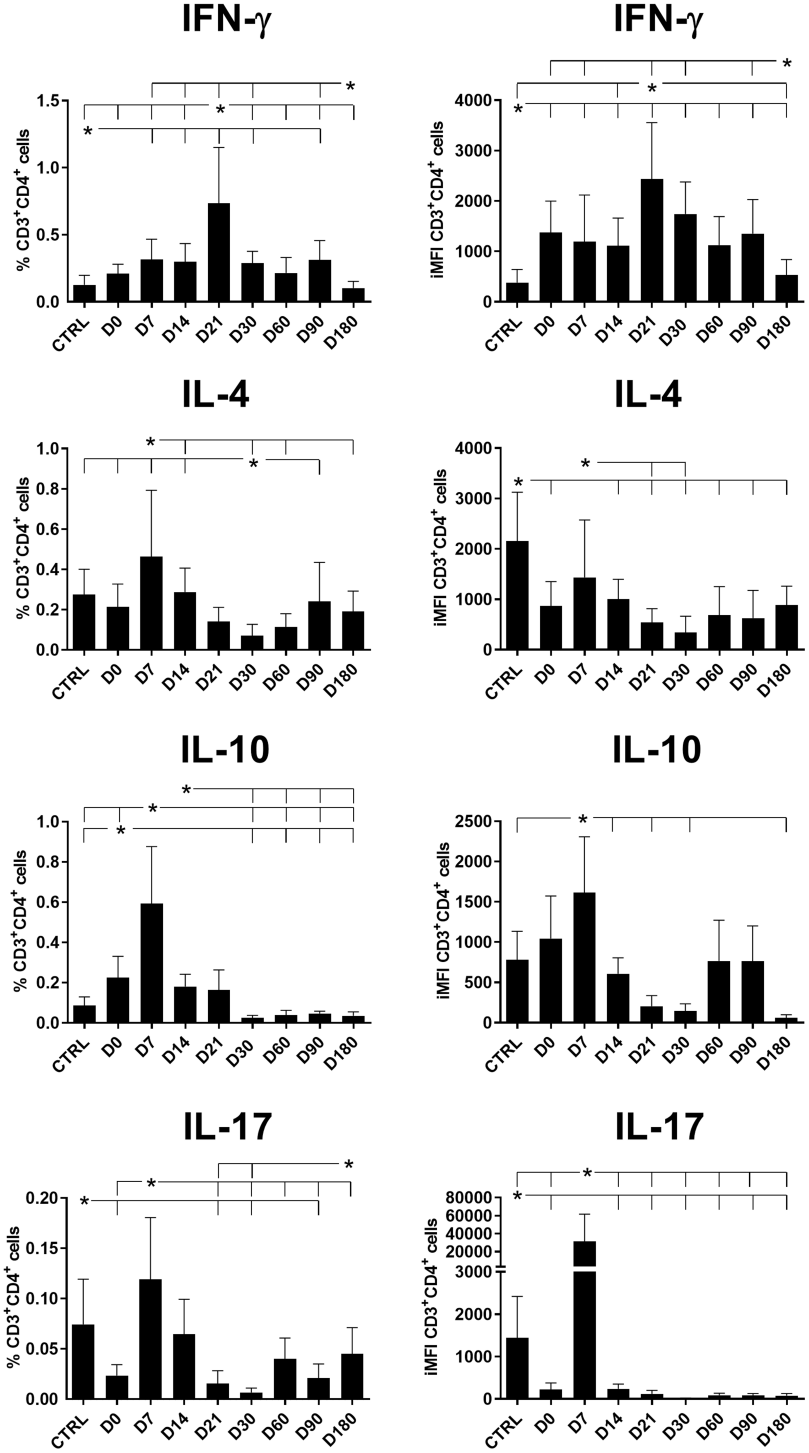
Total frequencies and iMFIs of cytokine-producing CD4^+^ T cells throughout treatment and follow-up for visceral leishmaniasis. Total frequencies (left column) and iMFI (right column) of IFN-γ, IL-4, IL-10, and IL-17 expressing CD4^+^ T cells in peripheral blood mononuclear cells (PBMCs) from healthy controls (CTRL) and patients with visceral leishmaniasis before treatment (D0) and during chemotherapy treatment follow-up. Results were subtracted from their background values of cells incubated without the soluble *Leishmania* antigen. The results in the panel are expressed as means + SE. Asterisks and horizontal lines indicate statistical differences as disclosed by 95% confidence interval (CI 95%) analysis.

The total frequencies and iMFI of IFN-γ-producing CD4^+^ T cells increased until the end of chemotherapy at day 21 (**Figure 1**). In contrast, the production of IL-4 and IL-10 in CD4^+^ T cells reached maximal values on day 7 and gradually decreased until day 30 (after the end of treatment). A difference was noted for IL-17 levels, which peaked on day 7 but rapidly diminished until day 21 in terms of the total frequencies and day 14 for iMFI values (**Figure 1**). The total frequencies of IFN-γ-producing and IL-17-producing CD4^+^ T cells were weakly but significantly positively correlated (*R* = 0.258, *p* = 0.036).

**Figure 2:**
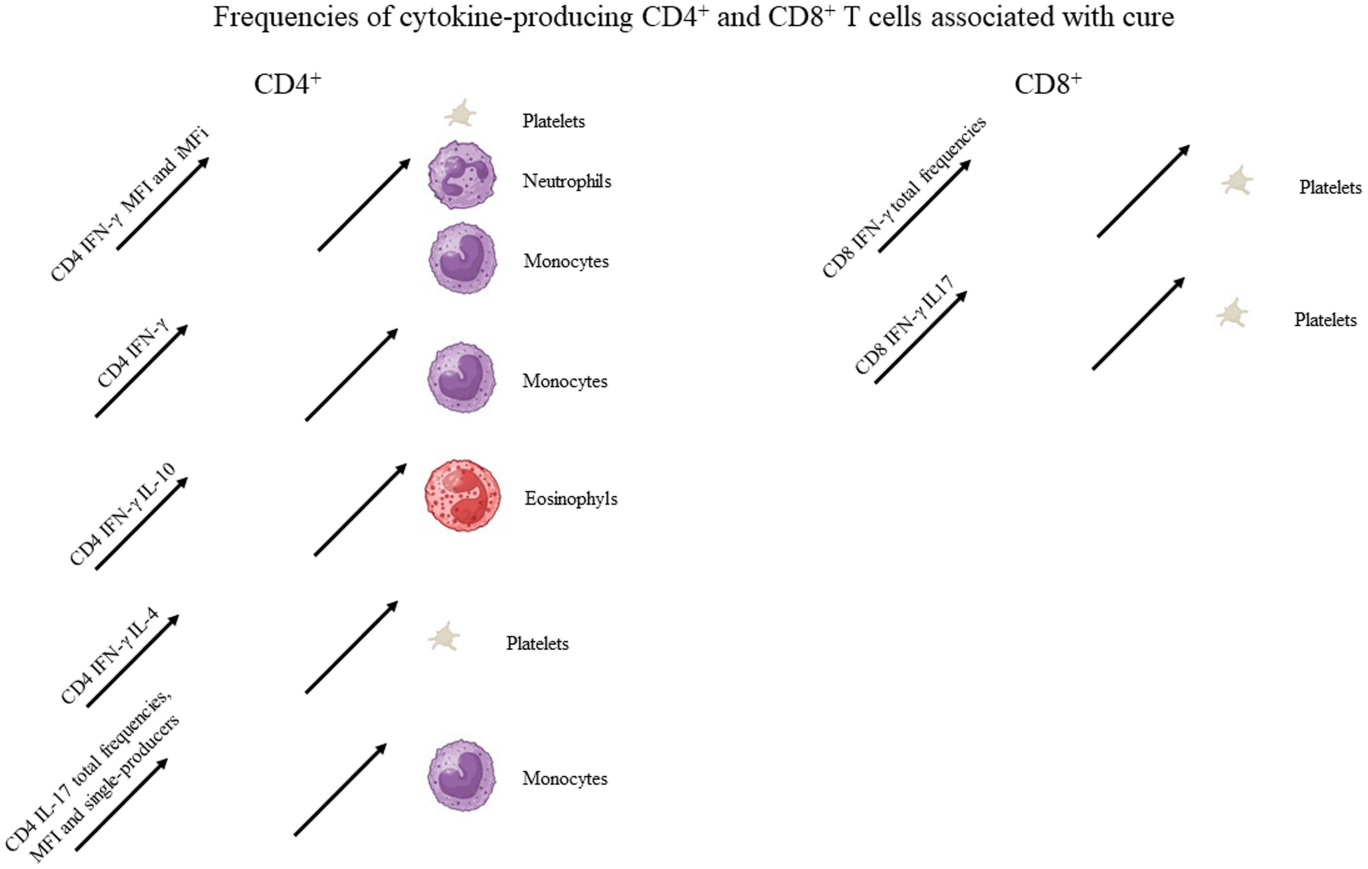
Graphical summary of the frequencies of cytokine-producing CD4 and CD8 T cells associated with cure. Increases in the frequencies of cytokine-producing T cells statistically correlated to the clinical variables associated with cure, as described in **Table 1**. For graphical representation of clinical outcomes, BioRender software was used.

**Figure 3:**
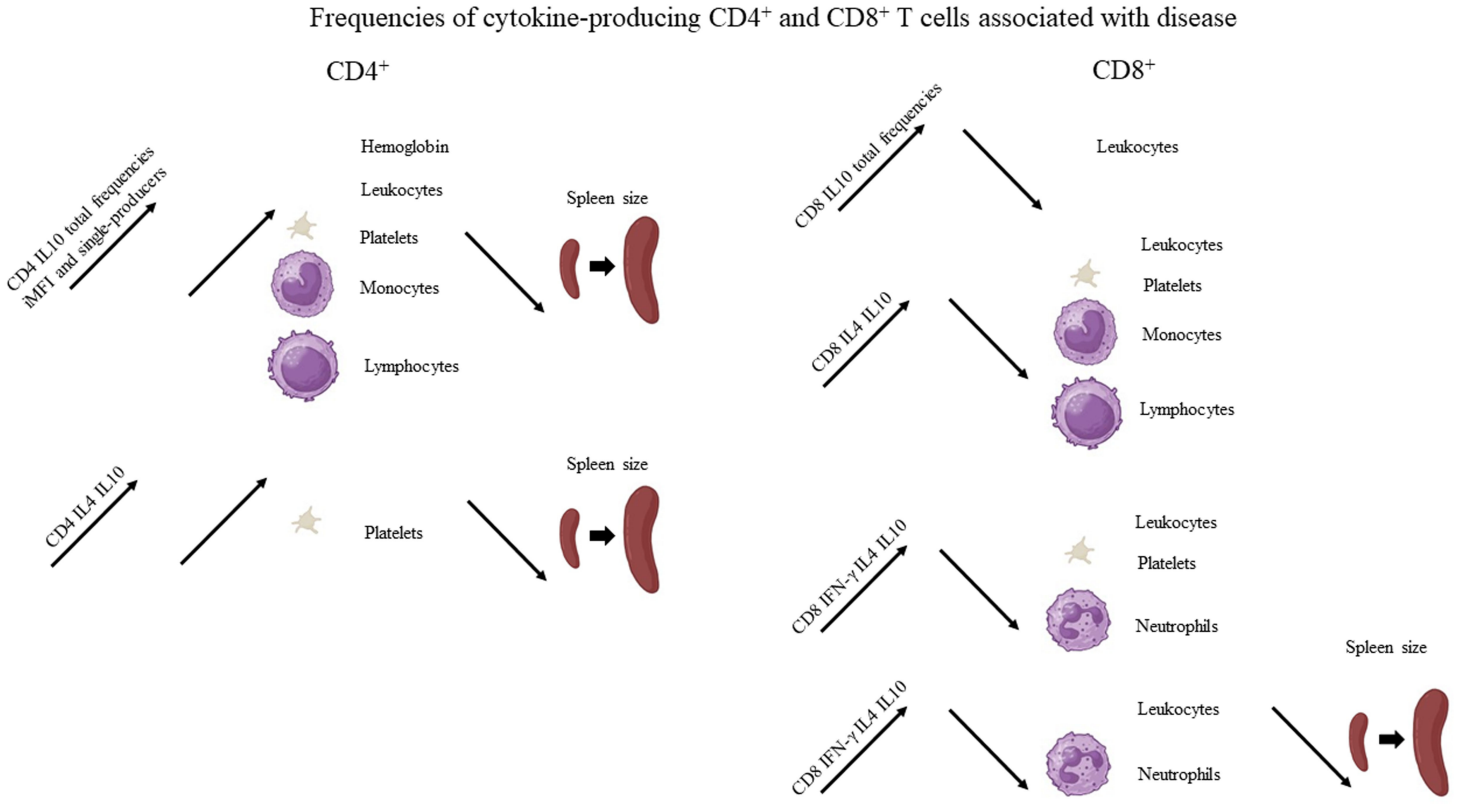
Graphical summary of the frequencies of cytokine-producing CD4 and CD8 T cells associated with disease. Increases of the frequencies of cytokine-secreting T cells statistically correlated to clinical variables associated with disease, as described in **Table 1**. For graphical representation of clinical outcomes, BioRender software was used.

In our study, the frequencies of CD4^+^ T cells producing IL-10, IL-4, and IL-17 reached their maximal values on day 7 while parasite replication was still active; however, the frequencies of these cells diminished after this point, which is when the chemotherapy likely started to impact the regulatory response and a Th1 response began to recover. Significant albeit weak positive correlations of the CD4^+^IFN-γ^+^ total frequencies and iMFIs with the level of monocytes and of the CD4^+^IFN^+^-γ MFIs with the platelet and neutrophil levels indicate their associations with the recovery of a Th1 response (**Table 1**, **Figure 2**). Conversely, the association of IL-10 production with a Th2 response was supported by the weak but significant (1) positive correlations of CD4^+^IL-10 total frequencies with spleen sizes; (2) negative correlations of CD4^+^IL-10^+^ total frequencies with hemoglobin (HB), platelet, and lymphocyte levels; and (3) negative correlations of CD4^+^IL-10^+^ iMFIs with HB, platelet, leukocyte, lymphocyte, and monocyte levels (**Table 1**, **Figure 3**).

**Table 1:** Correlations between clinical outputs and cytokine-producing CD4^+^ and CD8^+^ T-cell phenotypes.

T-cell phenotypes	Clinical outcomes	*R* values	*p* values
CD4^+^IFN-γ ^+^ total frequencies	Monocytes	0.3143	0.0054
CD4^+^IFN-γ ^+^ MFI	Platelets	0.3439	0.0022
	Neutrophils	0.2317	0.0426
CD4^+^IFN-γ^+^ iMFI	Monocytes	0.3851	0.0005
CD4^+^IL-10^+^ total frequencies	Spleen size	0.2188	0.0497
	HB	-0.2718	0.0168
	Platelets	-0.2282	0.0459
	Lymphocytes	-0.2923	0.0099
CD4^+^IL-10^+^ iMFI	HB	-0.2661	0.0193
	Platelets	-02264	0.0477
	Leukocytes	-0.2652	0.0198
	Lymphocytes	-0.2961	0.0089
	Monocytes	-0.2784	0.0142
CD4^+^IL-17^+^ total frequencies	Monocytes	0.3267	0.0037
CD4^+^IL-17^+^ MFI	Monocytes	0.2551	0.0252
CD4^+^IFN-γ ^+^IL-4^-^IL-10^-^IL17^-^	Monocytes	0.4977	<0.0001
CD4^+^IFN-γ ^-^IL-4^-^IL-10^+^IL17^-^	Spleen size	0.2773	0.0122
	Monocytes	-0.2339	0.0407
CD4^+^IFN-γ ^-^IL-4^-^IL-10^-^IL17^+^	Monocytes	0.4304	<0.0001
CD4^+^IFN-γ ^-^IL-4^+^IL-10^+^IL17^-^	Platelets	-0.2364	0.0385
CD4^+^IFN-γ ^+^IL-4^-^IL-10^+^IL17^-^	Eosinophils	0.2738	0.0313
CD4^+^IFN-γ ^+^IL-4^+^IL-10^-^IL17^-^	Platelets	0.2814	0.0132
CD8^+^IFN-γ ^+^ total frequencies	Platelets	0.2494	0.0333
CD8^+^IL-10 ^+^ total frequencies	Leukocytes	-0.2332	0.0471
CD8^+^IFN-γ ^-^IL-4^+^IL-10^+^IL17^-^	Platelets	-0.2408	0.0349
	Leukocytes	-0.2622	0.0213
	Lymphocytes	-0.2738	0.0160
	Monocytes	-0.2407	0.0350
CD8^+^IFN-γ ^+^IL-4^-^IL-10^-^IL17^+^	Platelets	0.2694	0.0178
CD8^+^IFN-γ ^+^IL-4^+^IL-10^+^IL17^-^	Platelets	-0.2317	0.0426
	Leukocytes	-0.2587	0.0231
	Neutrophils	-0.2601	0.0223
CD8^+^IFN-γ ^+^IL-4^+^IL-10^+^IL17^+^	Spleen size	0.2714	0.0143
	Leukocytes	-0.2599	0.0224
	Neutrophils	-0.2531	0.0264

Despite the observed decrease of CD4^+^IL-17^+^ total frequencies and iMFIs toward the cure of patients (**Figure 1**), both variables showed a weak but significant positive correlation with the level of monocytes (**Table 1**, **Figure 2**). It is worth noting that after cure, on day 180, a slight increase in CD4^+^IL-17^+^ total frequencies was observed (**Figure 1**).

### CD4 T cells producing one, two, three, or four cytokines

**Figure 4:**
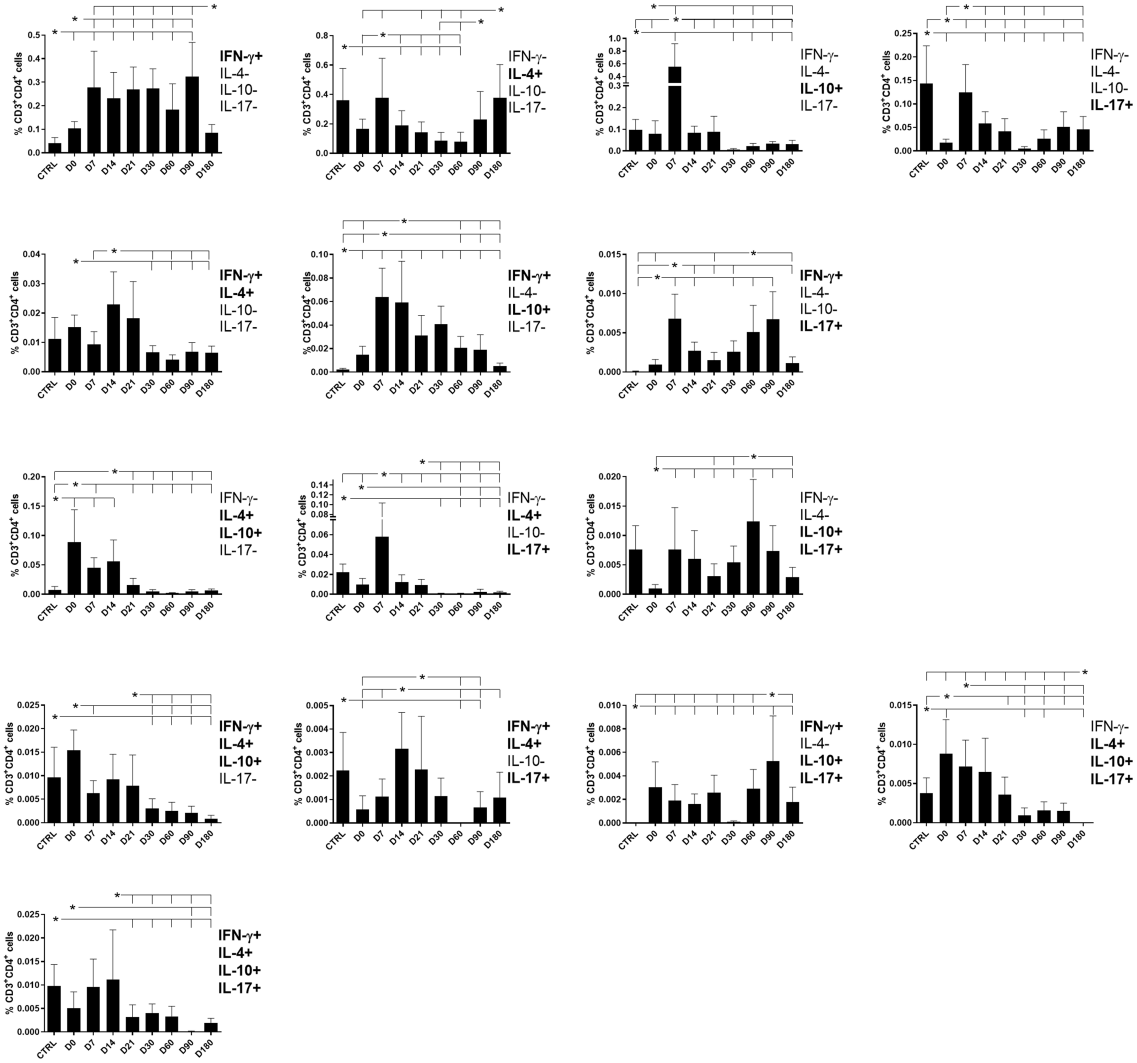
Different attributes of the CD4^+^ IFN-γ, IL-4, IL-10, and IL-17 T-cell response throughout treatment and follow-up for visceral leishmaniasis. Frequencies of CD4^+^ T cells producing one cytokine (IFN-γ, IL-4, IL-10, or IL-17) or a combination of two (IFN-γ^+^IL-10^+^, IFN-γ^+^IL-4^+^, IFN-γ^+^IL-17^+^, IL-4^+^IL-10^+^, IL-4^+^-IL-17^+^, or IL-10^+^IL-17^+^), three (IFN-γ^+^IL-4^+^IL-10^+^, IFN-γ^+^IL-4^+^IL-17^+^, IFN-γ^+^IL-10^+^IL-17^+^, or IL-4^+^IL-10^+^IL-17^+^), or four cytokines (IFN-γ^+^IL-4^+^IL-10^+^IL-17^+^) simultaneously during chemotherapy and treatment follow-up. Day 0 indicates the start of the treatment. CTRL indicates results from healthy controls. Results were subtracted from the background values of cells incubated without the soluble *Leishmania* antigen and are expressed as means + SE. Asterisks and horizontal lines indicate statistical differences as disclosed by 95% confidence interval (CI 95%) analysis.

CD4 T cells producing only one cytokine were largely more frequent than those producing two, three, or four (**Figure 4**). Among them, the proportions of CD4^+^ T cells producing only IFN-γ increased from day 7 and remained high until day 90 (**Figure 4**), suggesting the recovery of a Th1 response after treatment. Conversely, peaks of cells producing only IL-10, IL-4, and IL-17 were detected on day 7. However, while the frequency of IL-10-producing cells declined by day 14 and was almost undetectable after day 30, revealing the control of the Th2 response by the treatment, high frequencies of IL-4- and IL-17-producing CD4^+^ T cells were observed in the control and declined in samples from treated patients until day 60 and day 180, respectively. The frequencies of IL-4-producing CD4^+^ T cells notably increased on day 90 (**Figure 4**).

The frequencies of double-producers of IFN-γ and IL-4 and of IFN-γ and IL-10 also increased simultaneously from day 7 but declined by day 30 and day 60, respectively, while the frequencies of IFN-γ and IL-17 double-producers were increased on days 7, 60, and 90. In contrast, double-producers of IL-4 and IL-10 and of IL-4 and IL-17 were high on day 0 and decreased toward day 14 or day 21 indicating the control of the Th2 response after treatment. Producers of IL-10 and IL-17 showed peaks on day 7 and day 60 (**Figure 4**).

All frequencies of triple-producers, except for those that produced IFN-γ, IL10, and IL-17, were high in the control. The frequencies of triple-producers of IFN-γ, IL-4, and IL-10 and of IL-4, IL-10, and IL-17 declined over the treatment and follow-up period, while cells producing IFN-γ, IL-4, and IL-17 showed peaks on day 14 and day 21 and those producing IFN-γ, IL-10, and IL-17 peaked on day 90.

Finally, the proportions of CD4^+^ T cells producing all four cytokines were high since day 0 and declined after day 21, suggesting that they are more related to the pathology of VL (**Figures 3**, **4**).

Suggesting a potential association with a cure response, the frequencies of CD4^+^ T cells producing only IFN-γ or only IL-17 were correlated with the level of monocytes (**Table 1**, **Figure 2**). Interestingly, the double-producers of IFN-γ and IL-10 or IL-4 showed a significant albeit weak positive association with the levels of eosinophils and platelets, respectively (**Table 1**, **Figure 2**), suggesting the predominant role of IFN-γ in driving the immune response toward a Th1 response and cure. In contrast, suggesting association with the disease or Th2 response, (1) single-producers of IL-10 showed a weak but significant positive correlation with spleen sizes and a negative correlation with monocytes and (2) double-producers of IL-4 and IL-10 showed a weak but significant negative correlation with platelet levels (**Table 1**, **Figure 3**). Furthermore, no correlations were found between the clinical signs and frequencies of CD4^+^ T cells expressing three or four cytokines simultaneously (**Table 1**, **Figures 2**, **3**).

With regard to CD4^+^ cells, we concluded that the total frequencies, MFIs, iMFIs, and single-producers of IFN-γ, together with the double-producers of IFN-γ and IL-10 and of IFN-γ and IL-4, and total frequencies, MFIs, and single-producers of IL-17 are positively correlated with clinical variables that indicate VL cure (**Table 1**, **Figure 2**). The total frequencies, MFIs, and single-producers of IL-10 and double-producers of IL4 and IL-10 are negatively correlated to clinical outcomes associated with disease (**Table 1**, **Figure 3**).

### Total frequencies and iMFIs of cytokine-producing CD8^+^ T cells

**Figure 5:**
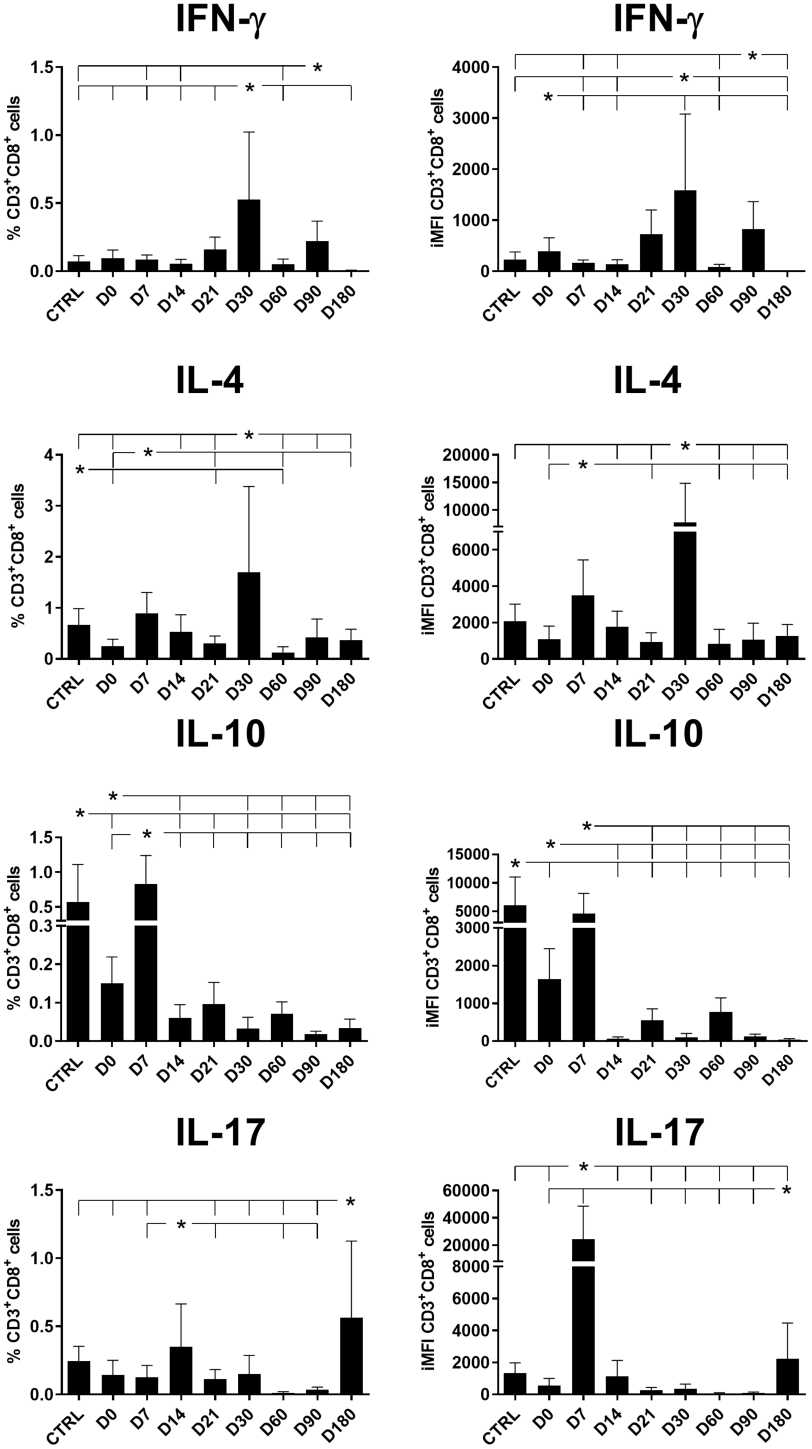
Total frequencies and iMFIs of cytokine-producing CD8^+^ T cells throughout treatment and follow-up for visceral leishmaniasis. Total frequencies (left column) and iMFIs (right column) of IFN-γ, IL-4, IL-10, and IL-17 expressing CD8^+^ T cells in peripheral blood mononuclear cells (PBMCs) from healthy controls (CTRL) and patients with visceral leishmaniasis before treatment (D0) and during chemotherapy and treatment follow-up. Results were subtracted from the background values of cells incubated without the soluble *Leishmania* antigen. The results in the panel are expressed as means + SE. Asterisks and horizontal lines indicate statistical differences as disclosed by 95% confidence interval (CI 95%) analysis.

The total frequencies and iMFIs of IFN-γ and IL-4-producing CD8^+^ T cells peaked on day 30, while CD8**^+^** T cells producing IL-10 and IL-17 peaked on day 7 and day 14, respectively, and decreased throughout the treatment and follow-up period (**Figure 5**), except for a slight increase only of IL-17-producing cells on day 180. In addition, the total frequencies of CD8**^+^** T cells producing IFN-γ were highly correlated to the frequencies of cells producing IL-4 (*R* = 0.8528; *p* < 0.0001) but not IL-10.

### CD8 T cells producing one, two, three, or four cytokines

**Figure 6:**
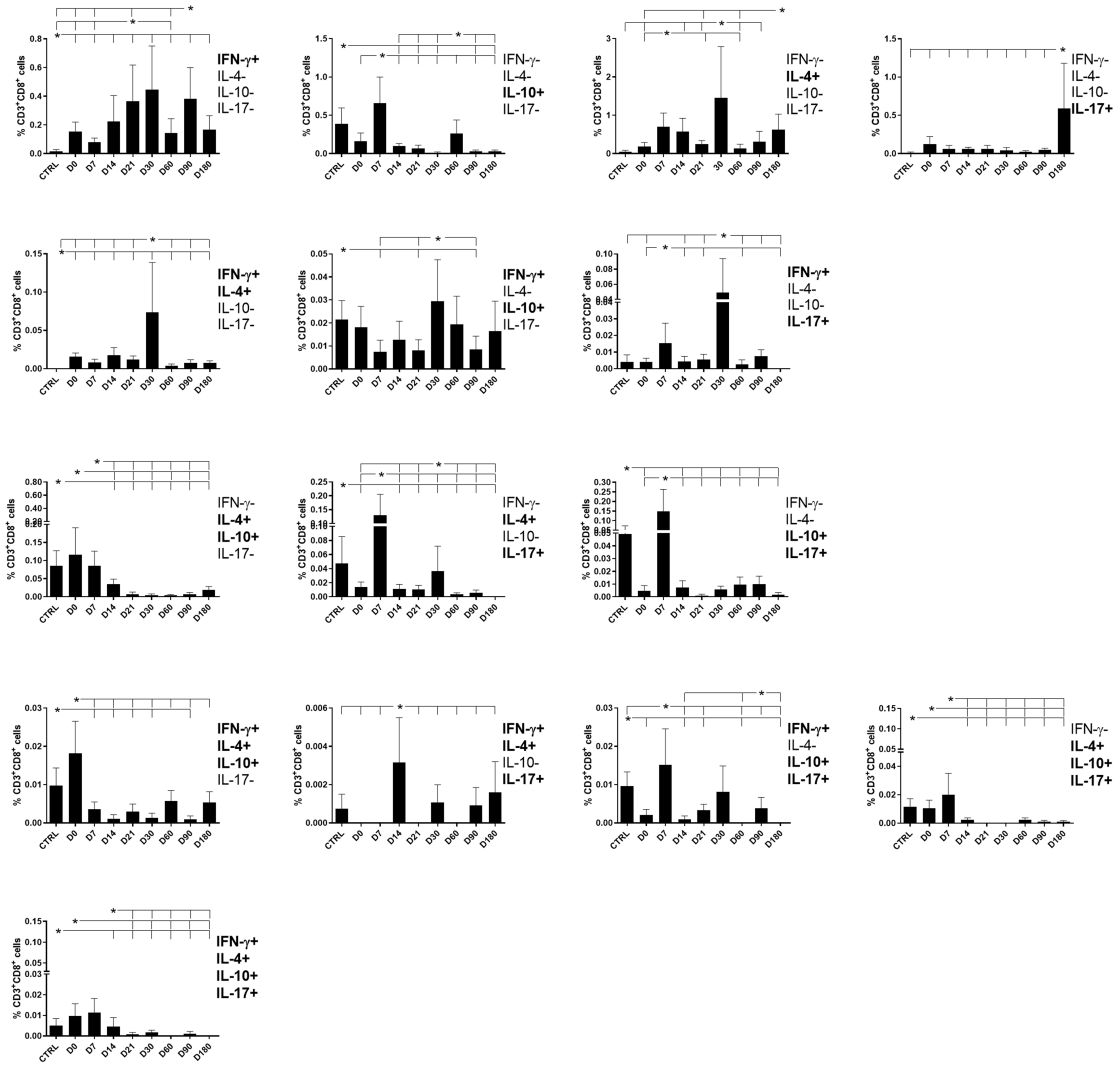
Different attributes of the CD8^+^ IFN-γ, IL-4, IL-10, and IL-17 T-cell response throughout treatment and follow-up of visceral leishmaniasis. Frequencies of CD8^+^ T cells producing one cytokine (IFN-γ, IL-4, IL-10, or IL-17) or combinations of two (IFN-γ^+^IL-4^+^, IFN-γ^+^IL-10^+^, IFN-γ^+^IL-17^+^, IL-4^+^IL-10^+^, IL-4^+^-IL-17^+^, or IL-10^+^IL-17^+^), three (IFN-γ^+^IL-4^+^IL-10^+^, IFN-γ^+^IL-4^+^IL-17^+^, IFN-γ^+^IL-10^+^IL-17^+^, or IL-4^+^IL-10^+^IL-17^+^), or four cytokines (IFN-γ^+^IL-4^+^IL-10^+^IL-17^+^) simultaneously during chemotherapy and treatment follow-up. Day 0 indicates the start of the treatment. CTRL indicates results from healthy controls. Results were subtracted from the background values of cells incubated without the soluble *Leishmania* antigen and are expressed as means + SE. Asterisks and horizontal lines indicate statistical differences as disclosed by 95% confidence interval (CI 95%) method.

As described for CD4^+^ T cells (**Figure 4**), CD8^+^ T cells producing only one cytokine were largely more frequent than those producing two, three, or four (**Figure 6**). The proportions of CD8^+^ T cells producing only IFN-γ increased from day 21 and remained high until day 90 (**Figure 6**), indicating that this population contributes VL cure. Conversely, a peak of cells producing only IL-10 was detected early, on day 7, and a decline was observed during the observation period, indicating that these cells are only present during disease. Furthermore, maximal proportions of IL-4-producing CD8^+^ T cells were detected on day 30 and of IL-17 on day 180.

The frequencies of CD8^+^ T cells producing IFN-γ in combination with IL-4, IL-10, or IL-17 reached maximal values on day 30 and decreased toward the end of the follow-up period, as was observed for single-producers of IFN-γ. This pattern indicates that the presence of IFN-γ expression, both in CD8^+^ T cells that are single- and double-producers, determines an immune response associated with the cure. In contrast, the frequencies of double-producers of IL-4 and IL-10, of IL-4 and IL-17, and of IL-10 and IL-17 started to decrease after day 0 or day 7, indicating that they are present during the disease and disappear after the cure (**Figure 6**).

The frequency pattern of CD8 lymphocytes expressing three or four cytokines was different from that of CD4 lymphocytes. The frequencies of triple- or quadruple-producers were high before treatment and decreased after the beginning of chemotherapy on day 0 (IFN-γ, IL-4, IL-10), on day 7 (IFN-γ, IL-10, IL-17, and IL-4, IL-10, IL-17), or on day 14 (IFN-γ, IL-4, IL-17 and IFN-γ, IL-4, IL-10, IL-17) (**Figure 6**). Our results indicate that CD8+ T cells producing three and four cytokines are more associated with the disease and disappear after the cure.

Supporting our observations (**Figure 6**), in association with the cure response, the total frequencies of CD8^+^ T cells producing only IFN-γ and double-producers of IFN-γ and IL-17 showed a weak but significant positive correlation with the platelet level (**Table 1**, **Figure 2**). This is in agreement with the increase of both types of cells up to day 30 (**Figure 6**). In contrast, as markers of disease, weak but significant negative correlations were found between (1) the total frequency of CD8^+^IL-10^+^ and the leukocyte level; (2) the frequency of double-producers of IL-4 and IL-10 and the platelet, leukocyte, lymphocyte, and monocyte levels; (3) the frequency of triple-producers of IFN-γ, IL-4 and IL-10 and the platelet, leukocyte, and neutrophil levels; and (4) the CD8 lymphocytes producing IFN-γ, IL-4, IL-10, and IL-17 and the leukocyte and neutrophil levels. Additionally, a weak but significant positive correlation was found between the frequency of CD8 lymphocytes producing IFN-γ, IL-4, IL-10, and IL-17 and the spleen size (**Table 1**, **Figure 3**). This is in accordance with the high proportions of these cells in untreated individuals, on day 0, and the subsequent decrease throughout the treatment and follow-up period (**Figure 6**).

We concluded that the total frequencies of CD8^+^ IFN-γ^+^ and CD8^+^ T cells that are double-secretors of IFN-γ and IL-17 are positively correlated with clinical variables that indicate the cure of VL (**Table 1**, **Figure 2**), whereas the total frequencies of CD8^+^ T cells producing IL-10, CD8^+^ T cells that are double-producers of IL-4 and IL-10, triple-producers of IFN-γ, IL-4, and IL-10, and producers of all four cytokines (IFN-γ, IL-4, IL-10, and IL-17) are negatively correlated to clinical outcomes associated with the disease (**Table 1**, **Figure 3**).

## Discussion

Chemotherapy for VL is limited to the use of a few licensed drugs, which show high toxicity (1, 30). A direct effect of chemotherapy drugs on the immune cells, irrespective of the pathogen impact, was recently observed (31–34). The experimental chemotherapy for VL with immucillins was shown to induce a Th1 response through a direct effect on T cells (31). Although IFN-γ secretion was high after immucillin treatment in infected animals, a lower but significant production of IFN-γ, TNF-α, and IL-10 and enhanced proportions of CD4^+^-T, CD19^+^ B, and CD8^+^ T cells were observed in uninfected animals treated with immucillins (31). Glucantime® was shown to have a lower and transient lymphoproliferative effect in this model (31). As has been suggested for Glucantime® and pentamidine, the efficacy of IA and IH immucillins is also partially T-cell dependent (35). Sodium antimony gluconate (SAG) has been demonstrated to activate the innate and adaptive immune system by indirectly triggering pathways for ROS and nitric oxide (NO) generation, imparting resistance to *Leishmania* infection and reinfection (33). Moreover, in a patient co-infected with mucocutaneous leishmaniasis and Chagas disease, MA treatment promoted a downmodulation of TNF-α, IL-6, and IL-10 cytokine levels but an upregulation of IL-12 production and a decrease of the frequency of circulating Treg, indicating the promotion of immune responses toward a more protective profile to both diseases (34). In addition, LAMB was shown to be immunosuppressive *in vitro* and on protection mediated by cytotoxic CD8 T cells in murine listeriosis. The dosage of 5 mg/kg body weight/day was also immunosuppressive in human therapy *in vivo* (32).

IFN-γ production has been widely associated with the cure of VL and is considered a sign of the successful recovery of a Th1 response (9, 11, 36–42). An increase in IL-10 production, on the other hand, is considered a marker of a Th2 response of overt VL (38–43). In experimental mouse models, IL-17 production has been associated to the influx of neutrophils to the infected organs and the control of parasite replication (26, 27), while in humans, the presence of this cytokine is linked to asymptomatic cases of VL (22). Furthermore, IL-17, in combination with IL-22 (44) or alone (45), has been demonstrated to have a role in protection and resistance against VL. However, other studies have associated IL-17 to VL susceptibility *via* the regulation of the IFN-γ response (46, 47).

In our study, IL-10, IL-4, and IL-17 reached their maximal values on day 7 after the beginning of treatment while parasite replication was still active; however, these frequencies diminished after this point when the chemotherapy started to impact the regulatory response of the disease and a Th1 response began to recover. The upregulation of IL-4, IL-17, and IL-10 and the loss of function of IFN-γ were considered as indicators of active disease, and IL-4 was initially regarded as a marker for active disease (42). With regard to IL-17 in our study, despite a decrease after the beginning of treatment, which indicated a potential correlation with a Th2 response and pathology, the total frequencies and iMFIs of CD4^+^IL-17^+^ T cells were positively associated to the level of monocytes, and the frequencies of single-producers of IL-17 and of double-producers of IFN-γ and IL-17 were increased on days 90 and 180, which was the defined point of cure, suggesting their association with a Th1 response. This is in agreement with the description of the involvement of IL-17 both in the proinflammatory anti-parasite effect (40, 48) and in pathology (40).

We further observed that the IFN-γ-producing CD4^+^ T cells’ total frequencies and iMFIs and the frequencies of CD4^+^ T cells that were single-producers or double-producers of IFN-γ, the latter in combination with either IL-10 or IL-4, increased throughout the treatment and follow-up period, thus becoming indicators of the recovery of a Th1 response. In agreement with this, the frequencies of double-producers of IFN-γ and IL-10 or of IFN-γ and IL-4 were positively associated with the levels of eosinophils and platelets, respectively, suggesting the predominant role of IFN-γ in leading to VL cure. These double-producers of IFN-γ, in combination with IL-10 or IL-4, may act as Tregs during treatment. Cells producing IFN-γ and IL-10 simultaneously, but not IFN-γ and IL-4, were described in patients that had recovered from VL as a regulatory subset of T cells important for maintaining a balance between Th1- and Th2-type cells in these individuals (49). A population of *Leishmania*-specific T cells’ double-producers of IL-10 and IFN-γ was also shown to expand in response to *L. donovani* infection (49). In addition and more in agreement with our results, the simultaneous production of IL-4 and IFN-γ by CD4^+^ and CD8^+^ cells was reported to be lower in patients with VL and higher in asymptomatic individuals than in healthy controls (50).

In patients with VL, monocytes/macrophages are cellular hosts to *Leishmania* parasites and ensure their survival by having an impaired oxidative burst and antigen presentation (51). Hematopoietic stem cells (HSCs) become activated after *Leishmania* bone marrow infection. In chronic VL, *L. donovani* induces HSC expansion and influences their differentiation toward progenitor cells of the regulatory phenotype, thus contributing to the pathogenesis of VL. Monocytes in the presence of soluble factors from infected bone marrow are more permissive to *Leishmania* infection (52). Furthermore, thrombocytopenia or less platelet generation has been associated to VL. In the initial phase, *Leishmania* visceral infection promotes platelet activation, which allows the recruitment of phagocytes and enhances the dissemination of the parasites. However, in the late infection, *Leishmania* modulates the stem lines, resulting in a marked thrombocytopenia and coagulation problems (53). In our study, CD4^+^ double-producers of IFN-γ and IL-10 or IFN-γ and IL-4 were positively correlated to eosinophil counts and potentially associated with VL cure. In agreement with this, the eosinophil percentage was high in the lymph nodes of *L. infantum*-infected dogs and the parasite load was negatively correlated with the eosinophil counts. Additionally, these dogs showed a high production of NO and ROS by eosinophils, suggesting that eosinophils may participate in antileishmanial immunity (54).

In our study, although the frequencies of CD4^+^double-producers of IFN-γ and IL-17 declined during treatment, a pronounced increase occurred on days 60 and 90, indicating their association with the recovered Th1 response. In contrast, in patients with tuberculosis, a double expression of IFN-γ and IL-17 by CD4^+^ T cells was correlated with the severity of the disease (21). To our knowledge, however, this is the first description of double-producers of IFN-γ and IL-17 in human patients of VL.

Regarding the Th2 response, IL-10 secretion is widely recognized as the hallmark of active VL (39, 40, 43, 55–58). In addition, IL-4 and IL-10 cytokines have been related to Treg, regulatory B cells, host susceptibility, and parasite persistence in VL (39). Although the involvement of multifunctional T cells producing double and triple combinations of IL-2, TNF-α, and IFN-γ in the generation of a Th1 response has been widely established in the vaccination and cure of leishmaniasis (9–11, 59), this approach has not been similarly explored regarding the double- and triple-producers of Th2 cytokines. In one study, a panel composed of IL-2, TNF-α, IFN-γ, and IL-10 was, however, set up in order to evaluate the influence of IL-10 on the quality and magnitude of the Th1 response after vaccination (60). In that study, inhibition of IL-10 at the time of immunization increased the magnitude of Th1 response and reduced the number of vaccine doses required (60).

In our study, the CD4^+^IL-10^+^ total frequencies and frequencies of CD4^+^ lymphocytes producing only IL-10 were readily detected in patients and reduced during the treatment and follow-up period, marking the control of the Th2 response by chemotherapy. In agreement and as expected for human VL, the frequencies of CD4^+^ T cell single-producers of only IL-10 were positively correlated with spleen sizes and negatively correlated with monocyte counts. The frequencies of double-producers of IL-4 and IL-10 likewise correlated negatively with platelet counts, suggesting their association with disease and a Th2 response. Moreover, the total frequencies of CD4-IL-10^+^ T cells correlated positively with spleen size and negatively with HB, platelet, and lymphocyte levels. In agreement with this, the iMFIs of CD4^+^IL-10^+^ T cells also correlated negatively with HB, platelet, leukocyte, lymphocyte, and monocyte levels, confirming that the CD4^+^ T cells producing IL-10 are markers of the disease, and the frequency of these cells correlates positively with its severity. Saha et al. (2007) described a direct positive correlation between the levels of IL-10 found in PBMC supernatants of post-kala-azar dermal leishmaniasis patients from India and the gravity and extension of their cutaneous lesions (43).

We observed that the frequency of CD4 cells’ double-producers of IL-4 and IL-10 and of IL-4 and IL-17 were high on day 0 and decreased toward the end of treatment and follow-up period, indicating that IL-4 is a marker of active disease. On the other hand, CD4+ double-producers of IL-10 and IL-17 showed a peak on day 7 and another on day 60 after treatment, probably revealing the importance of IL-17 in the recovery of a Th1 response.

Only the frequencies of CD4^+^ cells’ triple-producers of cytokines which express IL-17 showed a recovery on day 60, as it was observed for the total frequencies of CD4^+^IL-17^+^-secreting cells and for double-producers of IL-10 and IL-17. On the other hand, the frequencies of CD4^+^ producers of three or four cytokines simultaneously, that included IL-4, were high in patients with active VL, while it seems to prevent the increase promoted by IL-17 on day 60. No correlations were found between the clinical signs and frequencies of CD4^+^ T cells expressing three or four cytokines simultaneously.

The total frequencies and iMFIs of CD8^+^ T cells producing IFN-γ or IL-4 were low in patients with active VL and elevated only after chemotherapy, on day 30, while the total frequencies and iMFIs of CD8^+^ producers of IL-10 were only high in patients at the beginning of treatment, indicating their association with the disease. For IL-17, CD8^+^ cells producing this cytokine were enhanced at the beginning of chemotherapy and at day 180, as was detected for CD4^+^ lymphocytes.

The frequencies of CD8^+^ T cells producing only IFN-γ were enhanced between days 21 and 90, with a peak on day 30. The frequencies of producers of IL-4 were elevated on day 30 and of IL-17 on day 180, indicating their association with the end of chemotherapy and with the total recovery response after cure, respectively. CD8^+^ T cells producing IFN-γ were also described in response to specific *L.* (*L.*) *donovani* antigens, after VL cure (61). In contrast, in our study, CD8^+^ T cells secreting only IL-10 peaked on day 7 and decreased over the treatment and follow-up period. In agreement with our results, IFN-γ production by CD8 T cells and a decrease of IL-10 expression by both CD4 and CD8 T cells were observed in mice treated with liposomes containing MA (62). Furthermore, CD8^+^IFN-γ^+^ T cells correlated negatively and CD8^+^IL10^+^ cells correlated positively with the number of amastigotes in the spleens of these mice (63).

The common peak on day 30 observed for the frequencies of CD8^+^ double-producers of IFN-γ in combination with IL-4, IL-10, or IL-17, and in single-producers of IFN-γ suggests that the expression of IFN-γ during treatment contributes to the recovery of the CD8^+^ immune response associated with the cure. On the other hand, double-producers of IL-4 and IL-10 were high in patients with active VL, while double-producers of IL-4 and IL-17 and of IL-10 and IL-17 peaked on day 7 and started to wane from day 7 onwards, indicating that they are present at the beginning of the treatment only.

In our study, total frequencies of CD8^+^ T cells producing only IFN-γ and double-producers of IFN-γ and IL-17 were positively correlated with platelet levels, indicating their association VL cure. However, frequencies of CD8^+^ T cells triple and quadruple- cytokine-producers were not only greater in patients with active disease, decreasing during treatment, but they were also statistically correlated with clinical outcomes of VL, indicating that these multifunctional CD8^+^ cells are more associated with the pathology of VL.

It is not clear whether the role of CD8 T cells in VL is associated to protection, pathology, or immunosuppression (64). In experimental models of VL, CD8^+^ T cells develop multiple functions, including both cytotoxic activity and cytokine and chemokine secretion (65). Conversely, it has been suggested that they suffer anergy or exhaustion in VL, becoming unable to contribute with protection (64). In the cohort of patients studied in this investigation, the frequencies of CD8^+^ T cells’ single- and double-producers of IFN-γ correlated with cure, while the triple- and quadruple-producers were associated with VL pathology. Furthermore, in the same study cohort, we previously demonstrated that the CD8^+Low^ population was associated with active VL, while the CD8^+High^ population correlated with cure (9).

## Conclusions

Clinical improvements that indicate the cure of VL, such as increases in platelet, neutrophil, monocyte, and eosinophil counts, were positively associated to the rise of a Th1 response during treatment (CD4^+^ T cell total frequencies, MFIs, iMFIs, and single-producers of IFN-γ), to CD4^+^ double-producers of IFN-γ and IL-10 or IL-4, and additionally, to the increase of a Th-17 response, both of CD4^+^ and CD8^+^ T cells (CD4^+^ total frequencies, MFIs, and single-producers of IL-17, CD8^+^IFN-γ^+^ total frequencies, and CD8^+^ double-producers of IFN-γ and IL-17).

In contrast, clinical outcomes associated with VL, such as decreases in HB, platelet, leukocyte, lymphocyte, monocyte, and neutrophil counts and increases in spleen size, were negatively correlated to a Th2-response (CD4^+^ total frequencies, MFIs, and single-producers of IL-10), to CD4^+^ double-producers of IL4 and IL-10, and additionally to CD8^+^IL-10^+^ total frequencies, CD8^+^ T cells’ double-producers of IL4 and IL-10, CD8^+^ triple-producers of IFN-γ, IL-4 and IL-10, and CD8 ^+^ producers of the four cytokines (IFN-γ, IL-4, IL-10, and IL-17).

## Data availability statement

The raw data supporting the conclusions of this article will be made available by the authors, without undue reservation.

## Ethics statement

The studies involving humans were approved by the Research and Ethics Committee of the Federal University of Sergipe (UFS)-University Hospital, Aracaju, Sergipe State (SE), Brazil (CAAE 0162.0.107.000-09). A written informed consent was obtained from all patients before the study. This study was performed according to the standards established by the Declaration of Helsinki and followed the guidelines and regulations of the Brazilian National Council of Health resolution 196/96. The studies were conducted in accordance with the local legislation and institutional requirements. The participants provided their written informed consent to participate in this study.

## Author contributions

MF: Formal analysis, Writing – original draft, Investigation. LR: Investigation, Writing – original draft, Formal analysis. AB: Investigation, Writing – review & editing. GC: Investigation, Writing – review & editing. JA-S: Writing – review & editing, Formal analysis. AS: Writing – review & editing, Investigation. AJ: Writing – review & editing. CP-d-S: Writing – review & editing, Formal analysis, Funding acquisition, Writing – original draft. RA: Conceptualization, Resources, Funding acquisition, Writing – review & editing. CC: Conceptualization, Investigation, Resources, Writing – review & editing.

## Supplementary material

Supplementary Figure S1 Pre-treatment flow diagram. The asterisk indicates that the differential diagnosis of patients with visceral leishmaniasis and their treatment was carried out following the guidelines of the Brazilian Ministry of Health (29).

Supplementary Figure S2 Post-treatment flow diagram. The asterisk indicates that the criteria of visceral leishmaniasis cure followed the guidelines of the Brazilian Ministry of Health (29).

Supplementary Figure S3 Gating strategy for multi-parameter cytometry analysis of T-cell populations. CD4^+^ and CD8^+^ lymphocyte populations were selected, and the intracellular IFN-γ, IL-4, IL-10, and IL-17-cytokine expression was analyzed using the Boolean gate methodology to generate each combination of single-, double-, triple-, and quadruple-cytokine-producing T cells.
