# Supplementary figures and images for: CD4+ Th1 and Th17 responses and multifunctional CD8 T lymphocytes associated with cure or disease worsening in human visceral leishmaniasis
Source: Front Immunol. 2024 Feb 12;15:1277557. doi: 10.3389/fimmu.2024.1277557 (PMC10895669; doi:10.3389/fimmu.2024.1277557)

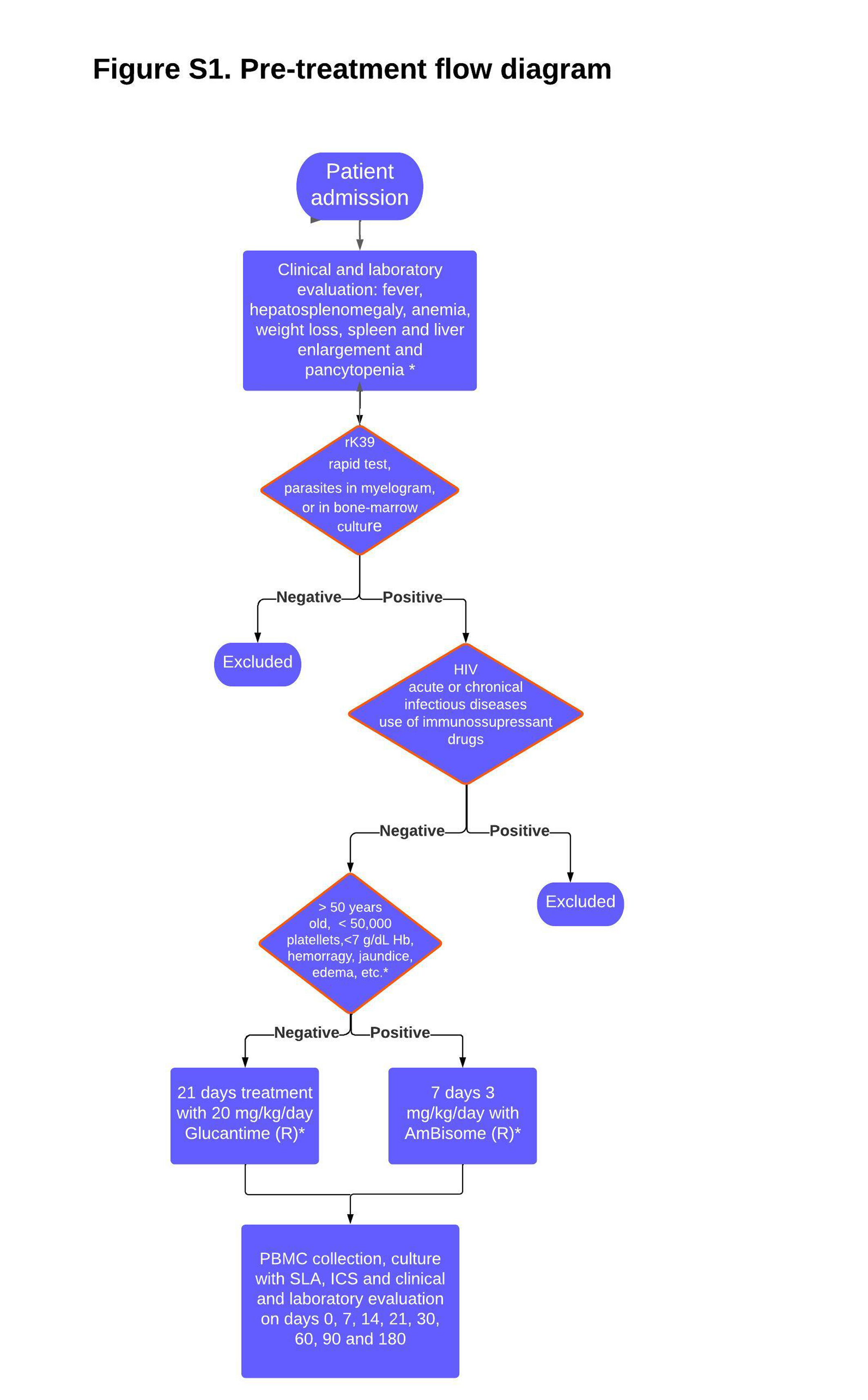

Supplement: Supplementary Figure S1 — Pre-treatment flow diagram. The asterisk indicates that the differential diagnosis of patients with visceral leishmaniasis and their treatment was carried out following the guidelines of the Brazilian Ministry of Health (29). [file Image_1.tif]

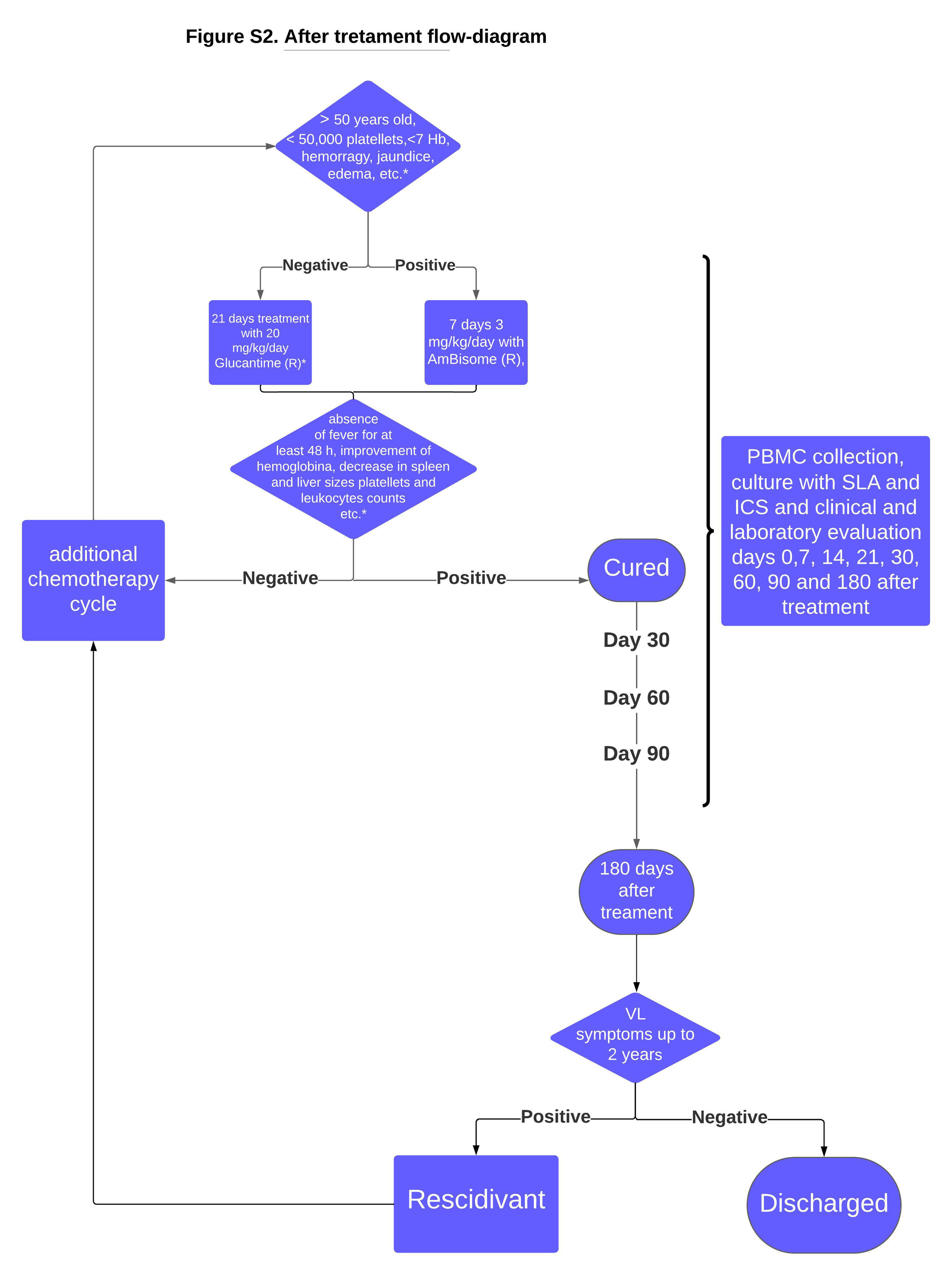

Supplement: Supplementary Figure S2 — Post-treatment flow diagram. The asterisk indicates that the criteria of visceral leishmaniasis cure followed the guidelines of the Brazilian Ministry of Health (29). [file Image_2.tif]

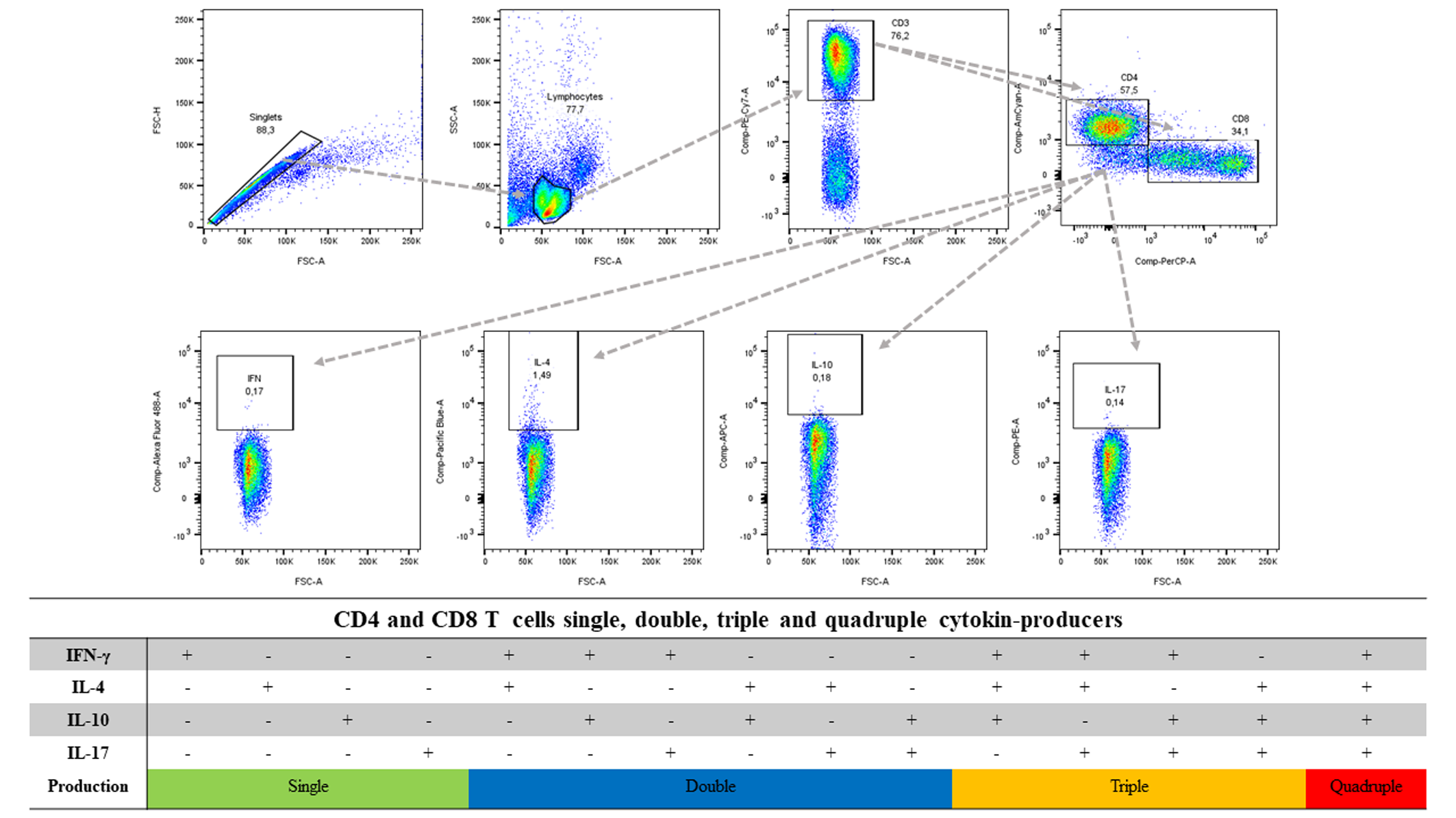

Supplement: Supplementary Figure S3 — Gating strategy for multi-parameter cytometry analysis of T-cell populations. CD4+ and CD8+ lymphocyte populations were selected, and the intracellular IFN-γ, IL-4, IL-10, and IL-17-cytokine expression was analyzed using the Boolean gate methodology to generate each combination of single-, double-, triple-, and quadruple-cytokine-producing T cells. [file Image_3.tif]
